# Tenofovir alafenamide nephrotoxicity: a case report and literature review

**DOI:** 10.1186/s12981-021-00380-w

**Published:** 2021-08-21

**Authors:** Thornthun Ueaphongsukkit, Sivaporn Gatechompol, Anchalee Avihingsanon, Jerasit Surintrspanont, Kroonpong Iampenkhae, Yingyos Avihingsanon, Suwasin Udomkarnjananun

**Affiliations:** 1 Department of Medicine, Faculty of Medicine, Chulalongkorn University, Bangkok, Thailand; 2 The HIV Netherlands Australia Thailand Research Collaboration (HIV-NAT), Thai Red Cross AIDS Research Centre, Bangkok, Thailand; 3 Tuberculosis Research Unit, Faculty of Medicine, Chulalongkorn University, Bangkok, Thailand; 4 Department of Pathology, Faculty of Medicine, Chulalongkorn University, Bangkok, Thailand; 5 Division of Nephrology, Department of Medicine, Faculty of Medicine, Chulalongkorn University and King Chulalongkorn Memorial Hospital, 1873, Rama 4 road, Pathumwan, 10330 Bangkok, Thailand; 6 Excellence Center for Solid Organ Transplantation, King Chulalongkorn Memorial Hospital, Bangkok, Thailand; 7 Renal Immunology and Transplant Research Unit, Chulalongkorn University, Bangkok, Thailand

**Keywords:** Tenofovir alafenamide, Acute kidney injury, Nephrotoxicity, Renal pathology, Mitochondria, HIV, Antiretroviral therapy, Case report

## Abstract

**Background:**

Tenofovir alafenamide (TAF), a novel prodrug of tenofovir (TFV), has become the preferred drug for the treatment of HIV-1 and chronic hepatitis B infection in clinical practice. Results from clinical trials showed that it had better renal and bone mineral outcomes compared to tenofovir disoproxil fumarate (TDF). However, as we have seen with TDF, side effects from the new medication can be more prevalent and recognized after extensive use in real world situations. Sporadic cases of acute kidney injury in patients using TAF have started to emerge.

**Case presentation:**

We report a case of 49-year-old Thai, HIV treatment-experienced female with hypertension presented with worsening renal function after switching her antiretroviral regimen from TDF, emtricitabine (FTC), and lopinavir/ritonavir (LPV/r) to TAF, FTC and dolutegravir (DTG) for 3 months. Kidney biopsy showed distinctive picture of tenofovir nephrotoxicity with acute tubular injury and mitochondrial injury. The possible causes of acute kidney injury and nephrotoxicity from TAF for this patient were discussed. We have extensively reviewed all published case reports of TAF-associated nephrotoxicity and summarized the essential information in this article.

**Conclusion:**

Although TAF has less nephrotoxicity compared with TDF; renal function should always be monitored after the initiation of both drugs. Future large cohort studies are required to identify the risk factors of TAF-associated nephrotoxicity and to design an effective preventive strategy.

## Introduction

Tenofovir (TFV) has become one of the backbone antiretroviral therapies (ART) in this era. However, the nephrotoxicity profile which is caused by cytoplasmic and intra-mitochondrial accumulation of TFV and results in the messenger ribonucleic acid (mRNA) depletion, mitochondrial deoxyribonucleic acid (DNA) depletion, and oxidative respiratory chain dysfunction, eventually contribute to proximal renal tubular abnormalities and renal insufficiency, limit its use in clinical practice [1, 2]. Tenofovir alafenamide (TAF) is a novel prodrug of TFV. It has a more favorable renal and bone safety profiles than its predecessor tenofovir disoproxil fumarate (TDF). Since 2015, TAF was approved by the U.S. Food and Drug Administration (FDA) as the first-line treatment of HIV in adults and adolescents. It is recommended as the preferred nucleotide analogue reverse transcriptase inhibitor (NRTI) backbone of the ART in the current HIV treatment guidelines [3, 4]. Since TAF has become more widely available, sporadic cases of acute kidney injury in patients using TAF is increasing [5–9]. Here we present a patient with TAF-containing ART regimen who came to our hospital with kidney injury.

## Case presentation

The patient was a 49-year-old Thai female with HIV infection, hypertension, and dyslipidemia. She was diagnosed with HIV infection since 1997 and had been exposed to multiple antiretroviral medications. In October 2001, she finally achieved undetectable viral load (< 50 copies/mL). In November 2015, she started to use TDF-based ART, which was TDF, emtricitabine (FTC), and lopinavir/ritonavir (LPV/r). Her serum creatinine and estimated glomerular filtration rate (eGFR) by CKD-EPI were stable at < 1 mg/dL and > 80 ml/min/1.73m^2^ since then.

In October 2019, her regimen was switched to a once daily, fixed dose combination pill containing TAF 25 mg, FTC 200 mg, and dolutegravir (DTG) 50 mg (TAF/FTC/DTG). Three months after she had changed her regimen (January 2020), serum creatinine increased from baseline of 1.05 mg/dL to 1.47 mg/dL. At 6 months follow-up (April 2020), her serum creatinine continuously increased to 2.30 mg/dL which prompted further investigation as described below.

Other concomitant medications included amlodipine 5 mg/day for hypertension and atorvastatin 20 mg/day for dyslipidemia. However, in January 2020, her hypertension was not well controlled and amlodipine was increased to 10 mg/day.

## Investigations

**Fig. 1:**
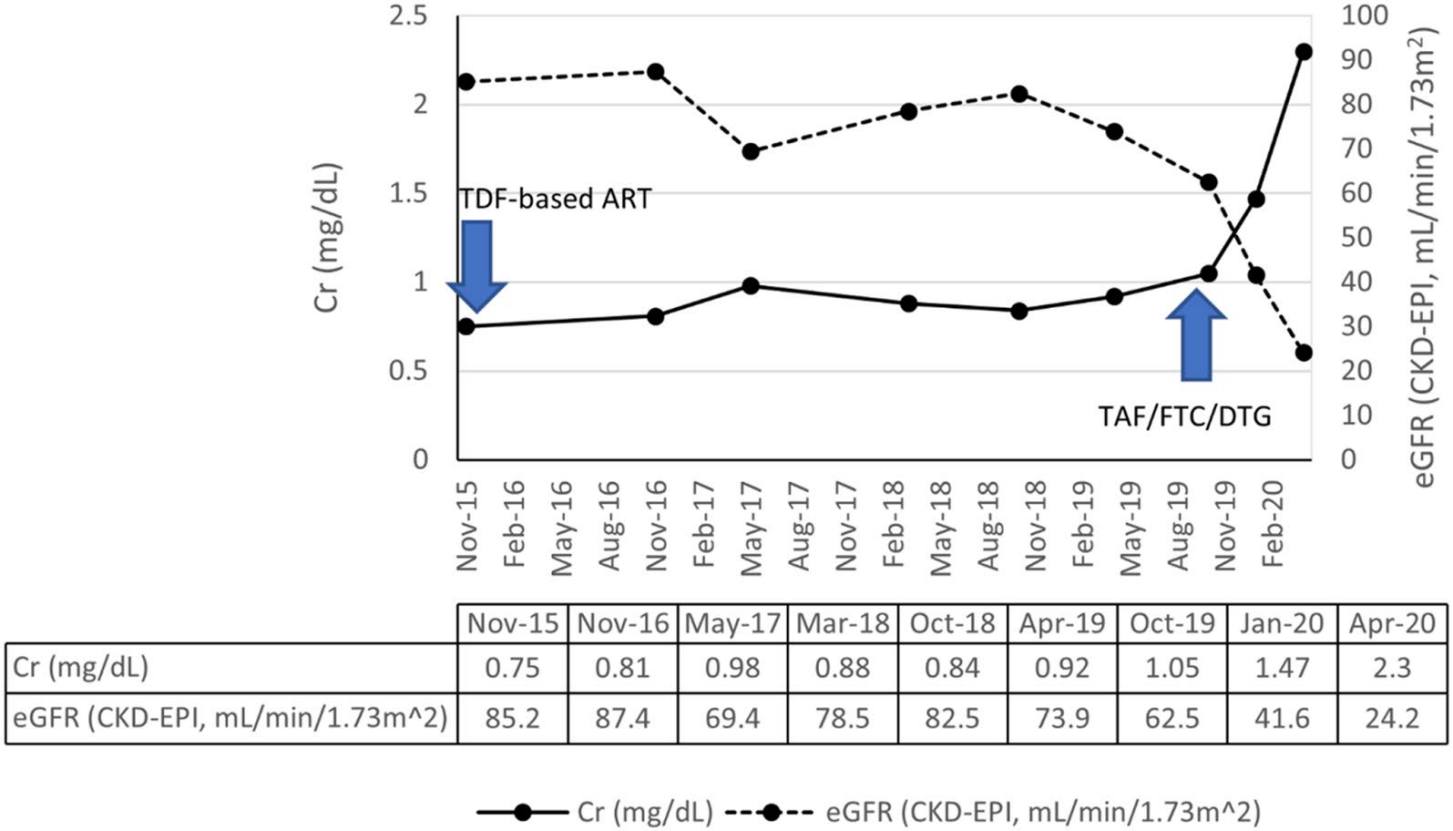
The patient’s serum creatinine and eGFR (CKD-EPI creatinine equation) before and after starting TAF/FTC/DTG

The patient’s baseline serum creatinine was within 0.8–1.0 mg/dL and eGFR (CKD-EPI) was within 70–85 mL/min/1.73m^2^ as shown in Fig. 1. Retrospective review of the medical record showed that she had persistent red blood cells (RBC) within the range of 3–5 cells/high power field (HPF) in urine and 1 + proteinuria for nearly eight years.

After starting of TAF/FTC/DTG, her serum creatinine was rapidly increased to 2.30 mg/dL and eGFR declined to 24 mL/min/1.73m^2^. Creatine phosphokinase (CPK) was within the normal range. Ultrasonography revealed a mild dilatation at the left pelvicalyceal system and mild hydronephrosis which could not be responsible for the significant deterioration of the patient’s renal function.

**Fig. 2:**
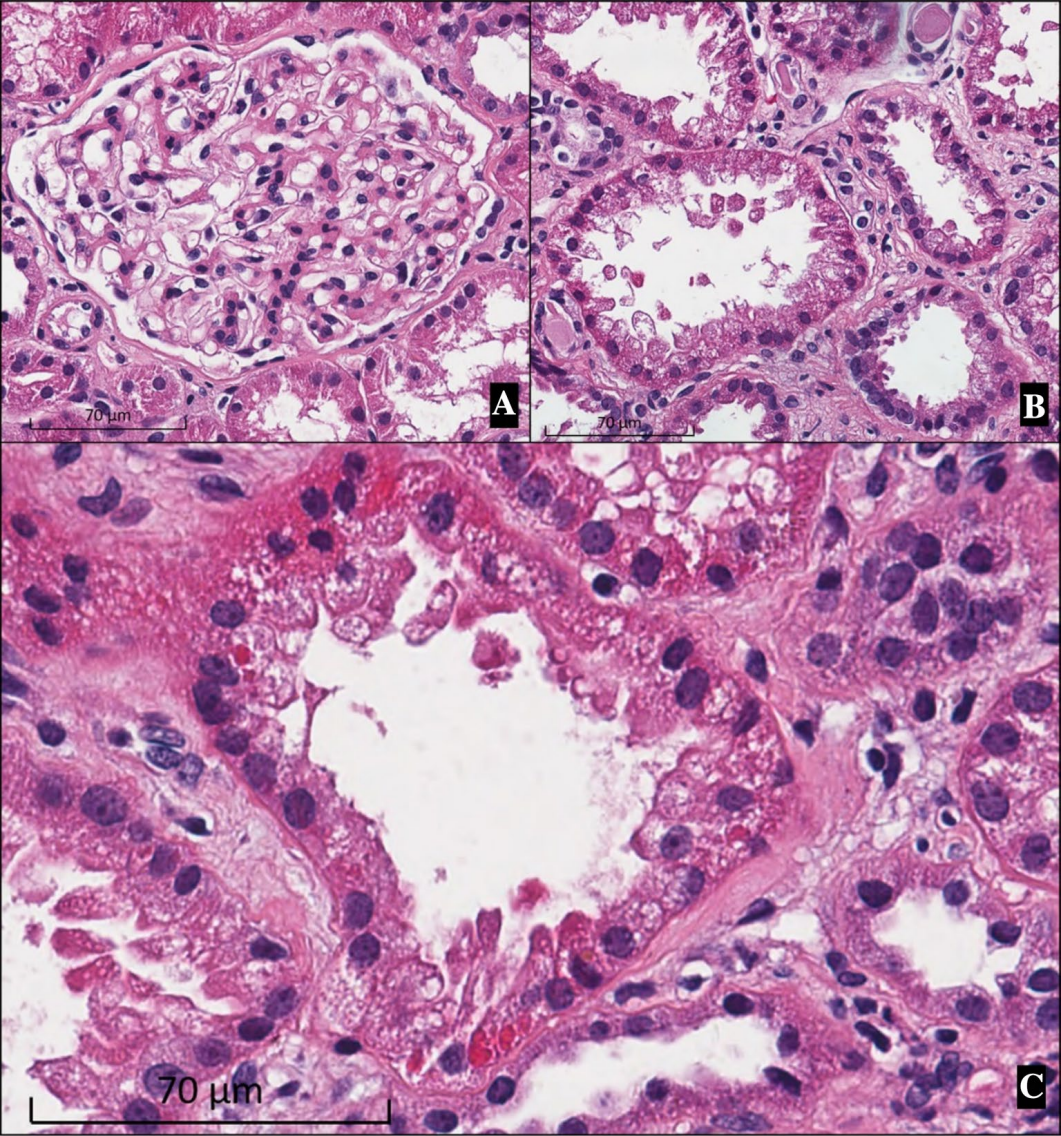
Kidney biopsy (hematoxylin and eosin staining, original magnification × 400). **A** A glomerulus with mild mesangial expansion and mesangial hypercellularity. **B** Acute tubular injury showing apical blebs, disruption of the brush border, apoptotic and sloughing of the renal tubular epithelial cells. **C** Megamitochondria is suspected from eosinophilic intracytoplasmic inclusions in the proximal tubular epithelial cells

A kidney biopsy was then performed. From the light microscopic examination of the specimen, glomeruli with mild mesangial expansion and mesangial hypercellularity were detected (Fig. 2A). Some tubules showed apical blebs and disrupted brush border (Fig. 2B). Apoptotic tubular cells were occasionally seen (Fig. 2B). Eosinophilic intracytoplasmic inclusions in the proximal tubular epithelial cells resembled to megamitochondria were identified in a few tubules (Fig. 2C). Immunofluorescence study showed granular staining of IgA 3 + and C3 1 + in the mesangium. The pathological findings were compatible with IgA nephropathy (Oxford classification M1 E0 S1 T1 C0) with acute tubular injury suspected to be from drug toxicity (tenofovir).

## Treatment and outcome

Since the patient’s renal function continuously declined, TAF/FTC/DTG was discontinued. Lopinavir/ritonavir and low dose lamivudine were prescribed thereafter. After TAF/FTC/DTG was discontinued for 2 months, her serum creatinine decreased to 1.82 mg/dL and eGFR improved from 24 to 32 mL/min/1.73m^2^. Her renal function was stabilized within the eGFR range of 35–40 mL/min/1.73m^2^ during the next 6 months. The patient had no hypophosphatemia or hypokalemia. After renal function was steady, amlodipine was switched to losartan to reduce proteinuria and control the blood pressure. Patient’s proteinuria was stable at 1.5–2 g/day during the follow-up periods after the initiation of losartan. Urine RBCs was persistently positive for 1–5 cells/HPF. After discussed with the patient regarding eGFR, proteinuria, and hypertension, kidney biopsy will be reperformed if these parameters are worsened, and corticosteroid is planned if there is a significant progression of IgA nephropathy.

## Discussion

This article presented a case report of the HIV-infected patient who had an acute kidney injury while receiving TAF/FTC/DTG. Her renal function had been stable for many years with the TDF-containing ART, before switching to the culprit regimen. Many possibilities are needed to be discussed of how her renal function was deteriorated.

First, the pathological findings suggested that the patient had a significant acute tubular injury which could have been caused by tenofovir toxicity. Tenofovir (TFV)-associated nephrotoxicity have long been described. It is more well-known with TDF which is a prodrug of TFV. TDF is converted to TFV in plasma and can damage the mitochondria of the proximal tubular cells [10]. Conversely, TAF is another TFV prodrug that is more stable in the plasma and is converted to TFV intracellularly [11]. Previous studies showed that therapeutic dose of TAF has a better renal safety profile compared with TDF, due to the fact that the level of TFV in the plasma is lower, hence lessens the exposure of proximal tubular epithelial cells to TFV [12–14]. However, TFV from TAF is still renally excreted and may cause tubular injury [15–17]. A pharmacokinetic study of a single-dose TAF administered to severe renal impairment participants with eGFR 15–29 mL/min/1.73m^2^ resulted in higher TAF and TFV plasma levels compared to the matched normal healthy controls with eGFR ≥ 90 mL/min/1.73m^2^ [18]. As a result, one can postulate that in patients with baseline renal insufficiency, the regular dose of TAF could result in higher plasma TFV level and lead to higher risk of kidney injury, compared with the normal eGFR patients. Case reports of TAF-associated nephrotoxicity were reviewed and shown in Table 1. [5–9]. Most of the cases had either underlying renal abnormalities or concurrent use of nephrotoxic medications. These renal diseases and co-medications possibly put the patients at risk of TFV-associated nephrotoxicity by decreasing TFV renal clearance [16]. In addition, renal diseases and medications could also be the causes of acute kidney injury by themselves. Our patient might have an underlying stable IgA nephropathy for a long period which did not display any problem while TDF had been used. Moreover, TDF was stopped for 3 months prior to the presentation and 8 months before the biopsy, while TAF was used at the time of presentation. Hence, this information supports the hypothesis that TAF, rather than TDF, were the cause of acute tubular injury in this case. However, we cannot completely exclude the possibility that the histopathology of TFV-associated nephrotoxicity was partly contributed from TDF before replaced by TAF [19, 20]. However, TDF solely cannot explain the clinical course of acute kidney injury in this patient.

**Table 1:** Case reports of acute kidney injury associated with tenofovir alafenamide use

Clinical feature	Novick TK et al. 2017 [9]	Serota DP et al. 2018 [8]	Alvarez H et al. 2018 [7]	Bahr NC et al. 2019 [6]	Heron JE et al. 2020 [5]	The present case
Age (years)	58	70	51	54	46	49
Sex	Male	Male	Male	Male	N/A	Female
Ethnic	African American	N/A	Caucasian	N/A	N/A	Asian
Co-infection	HCV	HCV	None	None	HCV	None
Co-morbidities	Cirrhosis Diabetes Drug abuse	Cirrhosis Alcohol use	None	Dyslipidemia Hypothyroidism	Hodgkin lymphoma	Hypertension Dyslipidemia IgA nephropathy
Viral load (copies/mL)	14,000	> 1 million	38	suppressed	N/A	< 50
CD4 (cells/µL)	367	100	587	N/A	N/A	1,096
TDF exposure	2 years	None	6 years	10 years	Yes	4 years
Baseline Cr (mg/dL)	0.9	1.2	N/A	0.59	N/A	1.05
Baseline CrCl	N/A	50 mL/min	N/A	N/A	N/A	63.5 mL/min/1.73m^2^
Baseline proteinuria	UPCI 0.27 g/gCr	N/A	N/A	N/A	N/A	Protein 2 +
Regimen	TAF/FTC + DRV/c	EVG/c/FTC/TAF	EVG/c/FTC/TAF (intentional overdose)	TAF/FTC + DRV/r + RAL	TAF + FTC + DRV/c	TAF/FTC/DTG
Significant concurrent medications	None	Sofosbuvir Ledipasvir	None	None	Carboplatin Gentamicin	None
Duration of TAF prior to presentation	2 months	3 months	9 months	2 months	N/A	3 months
Presentation	Oliguric acute kidney injury with volume overload	Acute kidney injury with hyperkalemia and non-anion gap metabolic acidosis	Acute kidney injury	Fanconi syndrome	Renal proximal tubulopathy (hypophosphatemia, hypokalemia, glucosuria, and proteinuria)	Acute kidney injury with proteinuria and hematuria
Cr at diagnosis (mg/dL)	4.0	5.2	2.15	5.56	0.76	2.30
Proteinuria	24-h urine protein 8.5 g/day	24-h urine protein 6.3 g/day	No proteinuria	N/A	UPCI 1.36 g/g	Protein 3 +
Urine sediments (cells/HPF)	RBC 5 WBC 1	RBC 3 WBC 2	N/A	N/A	N/A	RBC 5–10 WBC 3–5
Kidney biopsy	Diabetic nephropathy, focal glomerular hypercellularity, immune complex deposition, and mitochondrial injury	Not done	Not done	Not done	Not done	IgA nephropathy, and acute tubular injury with megamitochondria
Treatment	Acute dialysis, TAF-containing regimen was stopped	TAF-containing regimen and ledipasvir were stopped	TAF-containing regimen was stopped	TAF-containing regimen was stopped	Gentamicin was stopped	TAF-containing regimen was stopped
Outcome	Recovery (4 weeks after discharge, Cr was 0.9 mg/dL)	Recovery (12 weeks after discharge, Cr was 1.32 mg/dL)	Recovery (Cr returned to baseline level after 2 weeks)	Recovery (3 months after discharge, Cr was 1.11 mg/dL)	Recovery of tubulopathy	Improvement of Cr (2 months after TAF was discontinued, Cr was 1.82 mg/dL)
Possible explanations	TAF and comorbidities	Drug-drug interaction between Ledipasvir, cobicistat, and TAF	Drug overdose of cobicistat and TAF	TAF	Sepsis and lymphopenia leading to TAF and gentamicin toxicities	TAF and comorbidities

DRV/c: Darunavir/Cobicistat; DRV/r: Darunavir/Ritonavir; EVG/c/FTC/TAF: Elvitegravir/Cobicistat/Emtricitabine/Tenofovir alafenamide; RAL: Raltegravir; UPCI: Urine protein creatinine index

Second, DTG can inhibit organic cation transporter 2 (OCT2) at the basolateral membrane of the proximal renal tubular cells which mediates tubular uptake of creatinine, results in decreasing of creatinine clearance and rising of serum creatinine without changing the true glomerular filtration rate [21–23]. From previous studies, estimated decrease in creatinine clearance was approximately 10–14% from baseline, and increase of serum creatinine of less than 0.5 mg/dL [21, 24–27]. The serum creatinine are usually stabilized after 2–4 weeks of DTG initiation [25–27]. It is possible that DTG could partly contribute to the rising of serum creatinine in this patient. However, her renal function continued to decline beyond the threshold of DTG effect, thus it is unlikely that DTG was the sole cause of an acute kidney injury. However, there are still limited information of the pharmacokinetic and drug interaction between DTG and TAF in the Asian population. In a large randomized controlled trial of DTG and TAF conducted in African patients with creatinine clearance higher than 60 mL/min showed that the combination of DTG and TAF had minimal adverse effects [28]. However, pharmacogenomic of DTG and TAF in Asians might be different from Africans. It is possible that this combination might increase the plasma and intracellular TFV to the toxic levels. Additional pharmacokinetics studies of DTG in combination with TAF in Asian population are needed.

IgA nephropathy could have caused the patient’s abnormal urinary sediments and proteinuria for many years before this presentation. IgA nephropathy is the most common primary glomerular disease in Asia and has been associated with various inflammatory and infectious diseases, including HIV infection [29, 30]. This patient might develop IgA nephropathy since 2012 when she started having microscopic hematuria and proteinuria. The pathological findings based on the Oxford (MEST) classification; mesangial hypercellularity (M1), segmental glomerulosclerosis (S1), and tubular atrophy and interstitial fibrosis (T1) were all described as poor prognostic markers for IgA nephropathy [31–35]. Thus, IgA nephropathy could be partly responsible to renal function deterioration in our case. However, the improvement and stabilization of renal function after TAF discontinuation and before the initiation of losartan cannot be exclusively explained by IgA nephropathy.

There are also some limitations in our report. Although there was a pathological evidence of tubular injury from tenofovir from the kidney biopsy, our case did not have other laboratory abnormalities associated with proximal renal tubulopathy such as glucosuria, phosphaturia, hypophosphatemia, hypokalemia, or metabolic acidosis. However, certain group of patients can have tenofovir-associated nephrotoxicity without classical laboratory abnormalities as previously been reported [10, 36]. Since the kidney pathology showed both the characteristics of IgA nephropathy and tenofovir-nephrotoxicity, it is worth to keep in mind that the combination of both, rather than the single entity, had an effect on the patient’s renal function.

In conclusion, the temporal relationship of the event in our case suggested that the cause of acute kidney disease could be multifactorial. However, the evidence from kidney biopsy informed that the possibility of TAF-associated nephrotoxicity could not be omitted. Clinicians should be aware of this adverse effect of TAF, especially when the drug is prescribed in patients with an underlying renal disease. In these patients, renal function should be closely monitored after the initiation of TAF. Literatures review of TAF-associated nephrotoxicity are summarized in Table 1, which the general prognosis was good and renal function could spontaneously recover after the withdrawal of the medication.

## Data Availability

All data are presented in the manuscript. Additional data are available on reasonable request.

## Abbreviations

ART: Antiretroviral therapy
Cr: Creatinine
CrCl: Creatinine clearance
CPK: Creatine phosphokinase
DRV/c: Darunavir/Cobicistat
DRV/r: Darunavir/Ritonavir
DTG: Dolutegravir
EFV: Efavirenz
eGFR: Estimated glomerular filtration rate
EVG/c/FTC/TAF: Elvitegravir/Cobicistat/Emtricitabine/Tenofovir alafenamide
FTC: Emtricitabine
HPF: High power field
LPV/r: Lopinavir/ritonavir
OCT2: Organic cation transporter 2
RAL: Raltegravir
RBC: Red blood cells
TAF: Tenofovir alafenamide
TDF: Tenofovir disoproxil fumarate
TFV: Tenofovir
UPCI: Urine protein creatinine index
WBC: White blood cells
